# Supplementary material for: De novo analysis of bulk RNA-seq data at spatially resolved single-cell resolution
Source: Nat Commun. 2022 Oct 30;13:6498. doi: 10.1038/s41467-022-34271-z (PMC9618574; doi:10.1038/s41467-022-34271-z)
Supplement: Supplementary file 3 — Description of Additional Supplementary Files [file 41467_2022_34271_MOESM3_ESM.pdf]

Description of additional supplementary Information Files Document

Supplementary Data 1. Detailed information of datasets used in Bulk2Space. A collection of datasets including bulk RNA-seq, single-cell RNAseq, and spatially resolved transcriptomics used for the benchmarking, validation, and application of Bulk2Space. Each dataset was organized by dataset name, data type (bulk, single-cell, or spatial), species (human, mouse), tissue (brain, kidney, liver, etc.), technology (10X Visium, Drop-seq, RNAseq, etc.), data source (GEO series, Dryad, website, etc.), and reference.

Supplementary Data 2. Benchmarking experimental design for the deconvolution step (Step 1) of Bulk2Space with paired simulation data. The procedure of paired data simulations where bulk RNA-seq and single-cell reference were generated from same scRNAseq data. First, the scRNA-seq data were randomly separated into two parts, data_1, and data_2, respectively. For each simulation, three bulk datasets were generated from data_1 by changing the cell-type proportion and aggregating all scRNA-seq in data_1. The other dataset (data_2) was treated as singlecell reference.

Supplementary Data 3. Benchmarking experimental design for the deconvolution step (Step 1) of Bulk2Space with unpaired simulation data. The procedure of unpaired data simulations where bulk RNA-seq and single-cell reference were generated from different scRNAseq data of human pancreas. We randomly selected one as the single-cell reference and another dataset from different resources as bulk transcriptome data.

Supplementary Data 4. Benchmarking experimental design for the spatial mapping step (Step 2) of Bulk2Space with paired simulation data. The procedure of paired data simulations where single-cell transcriptomics and spatial reference were generated from same scRNA-seq data. First, the scRNA-seq data were randomly separated into two parts, data_1, and data_2, respectively. For each simulation, five spatial references with 100, 200, 500, 1000, and 5000 spots were simulated by randomly choosing 10 cells from data_1 for each spot. The other dataset (data_2) was treated as single cells to be mapped.

Supplementary Data 5. Benchmarking experimental design for the spatial mapping step (Step 2) of Bulk2Space with unpaired simulation data. Single-cell dataset and spatial reference were generated from different scRNA-seq data of human pancreas. We randomly selected one as the spatial reference and another from different resources as single cells to be mapped. For each simulation, five spatial references with 100, 200, 500, 1000, and 5000 spots were also simulated.
