# Supplementary material for: De novo analysis of bulk RNA-seq data at spatially resolved single-cell resolution
Source: Nat Commun. 2022 Oct 30;13:6498. doi: 10.1038/s41467-022-34271-z (PMC9618574; doi:10.1038/s41467-022-34271-z)
Supplement: Supplementary file 5 — Reporting Summary [file 41467_2022_34271_MOESM5_ESM.pdf]

nature portfolio

Corresponding author(s): Xiaohui Fan, Huajun Chen, Yue Gao

Last updated by author(s): Oct 2, 2022

Reporting Summary

Nature Portfolio wishes to improve the reproducibility of the work that we publish. This form provides structure for consistency and transparency in reporting. For further information on Nature Portfolio policies, see our Editorial Policies and the Editorial Policy Checklist.

Statistics

For all statistical analyses, confirm that the following items are present in the figure legend, table legend, main text, or Methods section.

n/a	Confirmed	
☐	☒	The exact sample size (*n*) for each experimental group/condition, given as a discrete number and unit of measurement
☐	☒	A statement on whether measurements were taken from distinct samples or whether the same sample was measured repeatedly
☐	☒	The statistical test(s) used AND whether they are one- or two-sided<br>*Only common tests should be described solely by name; describe more complex techniques in the Methods section.*
☐	☒	A description of all covariates tested
☐	☒	A description of any assumptions or corrections, such as tests of normality and adjustment for multiple comparisons
☐	☒	A full description of the statistical parameters including central tendency (e.g. means) or other basic estimates (e.g. regression coefficient) AND variation (e.g. standard deviation) or associated estimates of uncertainty (e.g. confidence intervals)
☐	☒	For null hypothesis testing, the test statistic (e.g. *F*, *t*, *r*) with confidence intervals, effect sizes, degrees of freedom and *P* value noted<br>*Give P values as exact values whenever suitable.*
☒	☐	For Bayesian analysis, information on the choice of priors and Markov chain Monte Carlo settings
☒	☐	For hierarchical and complex designs, identification of the appropriate level for tests and full reporting of outcomes
☐	☒	Estimates of effect sizes (e.g. Cohen's *d*, Pearson's *r*), indicating how they were calculated

Our web collection on statistics for biologists contains articles on many of the points above.

Software and code

Policy information about availability of computer code

Data collection	Illumina® Xten

Data analysis

The bulk2space package is available at https://github.com/ZJUFanLab/bulk2space/.

Specific package version used for analysis are as follows (python):
python==3.8.5
CUDA==11.0
deep-forest==0.1.5
easydict==1.9
numpy==1.19.2
pandas==1.1.3
scanpy==1.8.1
scikit-learn==1.0.1
scipy==1.5.2
torch==1.7.1
tqdm==4.50.2
Unidecode==1.3.0

Specific package version used for analysis are as follows (R):
Seurat 4.0.4 for scRNA-seq data/spatially resolved transcriptomics data analysis
ggplot2 3.3.5 for plotting

For manuscripts utilizing custom algorithms or software that are central to the research but not yet described in published literature, software must be made available to editors and reviewers. We strongly encourage code deposition in a community repository (e.g. GitHub). See the Nature Portfolio guidelines for submitting code & software for further information.

Data

Policy information about availability of data

All manuscripts must include a data availability statement. This statement should provide the following information, where applicable:

- Accession codes, unique identifiers, or web links for publicly available datasets
- A description of any restrictions on data availability
- For clinical datasets or third party data, please ensure that the statement adheres to our policy

The original data used in this paper can be accessed through the following links:
(1) single-cell RNA-seq data of the human blood: GEO accession: "GSE92495 [https://www.ncbi.nlm.nih.gov/geo/query/acc.cgi?acc=GSE92495]";
(2) single-cell RNA-seq data of the human brain: GEO accession: "GSE103723 [https://www.ncbi.nlm.nih.gov/geo/query/acc.cgi?acc=GSE103723]";
(3) single-cell RNA-seq data of the human kidney: GEO accession: "GSE121862 [https://www.ncbi.nlm.nih.gov/geo/query/acc.cgi?acc=GSE121862]";
(4) single-cell RNA-seq data of the human liver: GEO accession: "GSE124395 [https://www.ncbi.nlm.nih.gov/geo/query/acc.cgi?acc=GSE124395]";
(5) single-cell RNA-seq data of the human lung: GEO accession: "GSE130148 [https://www.ncbi.nlm.nih.gov/geo/query/acc.cgi?acc=GSE130148]";
(6) single-cell RNA-seq data of the mouse brain: GEO accession: "GSE60361 [https://www.ncbi.nlm.nih.gov/geo/query/acc.cgi?acc=GSE60361]";
(7) single-cell RNA-seq data of the mouse kidney: GEO accession: "GSE119531 [https://www.ncbi.nlm.nih.gov/geo/query/acc.cgi?acc=GSE119531]";
(8) single-cell RNA-seq data of the mouse lung: GEO accession: "GSE127465 [https://www.ncbi.nlm.nih.gov/geo/query/acc.cgi?acc=GSE127465]";
(9) single-cell RNA-seq data of the mouse pancreas: GEO accession: "GSE84133 [https://www.ncbi.nlm.nih.gov/geo/query/acc.cgi?acc=GSE84133]";
(10) single-cell RNA-seq data of the mouse testis: GEO accession: "GSE112393 [https://www.ncbi.nlm.nih.gov/geo/query/acc.cgi?acc=GSE112393]";
(11) single-cell RNA-seq data of the human pancreas with 975 cells: GEO accession: "GSE81076 [https://www.ncbi.nlm.nih.gov/geo/query/acc.cgi?acc=GSE81076]";
(12) single-cell RNA-seq data of the human pancreas with 2133 cells: GEO accession: "GSE85241 [https://www.ncbi.nlm.nih.gov/geo/query/acc.cgi?acc=GSE85241]";
(13) single-cell RNA-seq data of the human pancreas with 597 cells: GEO accession: "GSE86469 [https://www.ncbi.nlm.nih.gov/geo/query/acc.cgi?acc=GSE86469]";
(14) four sets of single-cell RNA-seq data of the human pancreas with 1635, 1562, 3330, 1230 cells, respectively: GEO accession: "GSE84133 [https://www.ncbi.nlm.nih.gov/geo/query/acc.cgi?acc=GSE84133]";
(15) single-cell RNA-seq data of the human pancreas with 2288 cells (https://www.ebi.ac.uk/arrayexpress/experiments/E-MTAB-5061/);
(16) single-cell RNA-seq data of the mouse hypothalamus using 10X Genomics: GEO accession: "GSE113576 [https://www.ncbi.nlm.nih.gov/geo/query/acc.cgi?acc=GSE113576]";
(17) single-cell RNA-seq data of the mouse hypothalamus using Drop-seq: "GSE87544 [https://www.ncbi.nlm.nih.gov/geo/query/acc.cgi?acc=GSE87544]";
(18) three sets of single-cell RNA-seq data of the mice liver: GEO accession: "GSE119340 [https://www.ncbi.nlm.nih.gov/geo/query/acc.cgi?acc=GSE119340]";
(19) single-cell RNA-seq data of the mouse hippocampus region (https://www.dropbox.com/s/cs6pii5my4p3ke3/mouse_hippocampus_reference.rds?dl=0);
(20) two sets of single-cell RNA-seq data of the human pancreatic ductal adenocarcinoma (PDAC): GEO accession: "GSE111672 [https://www.ncbi.nlm.nih.gov/geo/query/acc.cgi?acc=GSE111672]";
(21) single-cell RNA-seq data of the human melanoma: GEO accession: "GSE72056 [https://www.ncbi.nlm.nih.gov/geo/query/acc.cgi?acc=GSE72056]";
(22) single-cell RNA-seq data of the mouse cortex region (https://www.dropbox.com/s/dl/cuowvm4vrf65pvq/allen_cortex.rds);
(23) single-cell RNA-seq data of the human prostate cancer (https://singlecell.broadinstitute.org/single_cell/study/SCP1415);
(24) MERFISH data of the mouse hypothalamic preoptic region at bregma 0.26 (https://datadryad.org/stash/dataset/doi:10.5061/dryad.8t8s248);
(25) slide-seq v2 data of the mouse hippocampus region (https://singlecell.broadinstitute.org/single_cell/study/SCP815/highly-sensitive-spatial-transcriptomics-at-near-cellular-resolution-with-slide-seqv2);
(26) three sets of spatially resolved transcriptomics data of the human PDAC using "Spatial Transcriptomics": GEO accession: "GSE111672 [https://www.ncbi.nlm.nih.gov/geo/query/acc.cgi?acc=GSE111672]";
(27) two sets of spatially resolved transcriptomics data of the human melanoma using "Spatial Transcriptomics" (https://www.spatialresearch.org/resources-published-datasets/doi-10-1158-0008-5472-can-18-0747/);
(28) two sets of 10X Visium data of the mouse anterior cortex and posterior cortex regions (https://www.10xgenomics.com/cn/resources/datasets/mouse-brain-serial-section-1-sagittal-anterior-1-standard-1-1-0);
(29) two sets of spatially resolved transcriptomics data of the human prostate cancer using "Spatial Transcriptomics" (https://www.spatialresearch.org/resources-published-datasets/10-1038-s41467-018-04724-5/);
(30) three sets of bulk RNA-seq data of the mice liver: GEO accession: "GSE119340 [https://www.ncbi.nlm.nih.gov/geo/query/acc.cgi?acc=GSE119340]";
(31) two sets of bulk RNA-seq data of the human PDAC: GEO accession: "GSE171485 [https://www.ncbi.nlm.nih.gov/geo/query/acc.cgi?acc=GSE171485]"50;
(32) bulk RNA-seq data of the human prostate cancer (https://portal.gdc.cancer.gov).
The bulk RNA-seq data of the mouse cortex and mouse hypothalamus regions reported in this manuscript using our in-house developed Spatial-seq have been

deposited to the Gene Expression Omnibus under accession number "GSE192999 [https://www.ncbi.nlm.nih.gov/geo/query/acc.cgi?acc=GSE192999]". All source data are provided with this paper.

Human research participants

Policy information about studies involving human research participants and Sex and Gender in Research.

Reporting on sex and gender	Reporting on sex and gender is not relevant to our study.
Population characteristics	Population characteristics is not relevant to our study.
Recruitment	Recruitment is not relevant to our study.
Ethics oversight	Ethics oversight is not relevant to our study.

Note that full information on the approval of the study protocol must also be provided in the manuscript.

Field-specific reporting

Please select the one below that is the best fit for your research. If you are not sure, read the appropriate sections before making your selection.

☒ Life sciences ☐ Behavioural & social sciences ☐ Ecological, evolutionary & environmental sciences

For a reference copy of the document with all sections, see nature.com/documents/nr-reporting-summary-flat.pdf

Life sciences study design

All studies must disclose on these points even when the disclosure is negative.

Sample size	Three C57BL/6J mice with the SPF level weighted from 20 to 25 g were applied to Spatial-seq, a multiplexed spatial RNA-seq protocol based on the laser capture microdissection (LCM) and the spatial barcoding strategy. A total of 214 isolated bulk tissue of 13 main brain regions from 25 coronal and 11 sagittal slices were captured and labeled by unique spatial barcodes. The slices covered the entire mouse brain, with all main regions being isolated from each slice and each region containing at least 3 samples, so there was no sample size calculation performed.<br><br>Four publicly available datasets were also analyzed in this study, including human pancreatic ductal adenocarcinoma (PDAC), human melanoma, mouse hypothalamus, and human prostate cancer.
Data exclusions	No data is excluded
Replication	For mouse experiments, three animals were processed independently on the same day and all experiments were successful with the minimum number of detected genes of 8211 and the maximum number of detected genes of 11363 for different regions.
Randomization	Randomization is not relevant to our study because we isolated all regions from each slices across the entire mouse brain.
Blinding	Blinding is not relevant to our study because we didn't include any comparison between different mice.

Reporting for specific materials, systems and methods

We require information from authors about some types of materials, experimental systems and methods used in many studies. Here, indicate whether each material, system or method listed is relevant to your study. If you are not sure if a list item applies to your research, read the appropriate section before selecting a response.

Materials & experimental systems

n/a	Involved in the study	
☒	☐	Antibodies
☒	☐	Eukaryotic cell lines
☒	☐	Palaeontology and archaeology
☐	☒	Animals and other organisms
☒	☐	Clinical data
☒	☐	Dual use research of concern

Methods

n/a	Involved in the study	
☒	☐	ChIP-seq
☒	☐	Flow cytometry
☒	☐	MRI-based neuroimaging

March 2021

Animals and other research organisms

Policy information about studies involving animals; ARRIVE guidelines recommended for reporting animal research, and Sex and Gender in Research

Laboratory animals	Three wild-type adult C57BL/6J mice (SPF, male, 20-25 g) aged 8-10 weeks were used for Spatial-seq experiments.
Wild animals	Wild animals are not involved in this study.
Reporting on sex	Only male mice were used in this study.
Field-collected samples	Field-collected samples are not involved in this study.
Ethics oversight	All animal experiments involved in this manuscript were approved by the Animal Experimentation Ethics Committee of Zhejiang University. Every effort was made to avoid pain or discomfort in the animals during the experiments and to minimize the number of experimental animals.

Note that full information on the approval of the study protocol must also be provided in the manuscript.
